# Diagnostic microarray test for a genetic predisposition to hereditary nonpolyposis colorectal cancer (HNPCC)

**DOI:** 10.1186/1897-4287-10-S4-A23

**Published:** 2012-12-10

**Authors:** M Kaszuba, A Sikorski, J Odważna, J Wojciechowicz

**Affiliations:** 1 Centrum Badań DNA, Poznań, Poland

## 

Colorectal cancer (CC), due to its frequency and social costs is a subject of extensive screening programs (colonoscopy and new developments) in several countries, including Poland. Relatively common and defined genetic predispositions to different forms of CC make genetic screening an attractive alternative, not only in families with positive CC history. Optimal testing strategy should be acceptable for subjects, high-throughput and cost-effective in mutation detection.

CBDNA Ltd. Company has developed and validated prototype diagnostic microarray test for genetic predisposition to nonpolyposis colorectal cancer (HNPCC). The test includes 169 selected mutations (point mutations and short insertions/deletions) in 5 genes: *MLH1*, *MSH2*, *MSH6*, *CHEK2* and *NOD2*. Total of 36 patients with CC, six of them fulfilling modified Amsterdam criteria for HNPCC, and the rest with less evident familial aggregation of cancers were included in the pilot study. Altogether 6 different mutations in 9 patients were identified (25%) within the group.

The effectiveness of genetic screening using the microarray method was similar to other molecular, but more expensive and time-consuming methods.

